# Therapeutic effects of neuregulin-1 in diabetic cardiomyopathy rats

**DOI:** 10.1186/1475-2840-10-69

**Published:** 2011-07-29

**Authors:** Bingong Li, Zeqi Zheng, Yunfeng Wei, Menghong Wang, Jingtian Peng, Ting Kang, Xin Huang, Jian Xiao, Yong Li, Zhe Li

**Affiliations:** 1 Department of Cardiology, First Affiliated Hospital, Nanchang University, Nanchang 330006, China; 2 Jiangxi Institute of Hypertensive Diseases, Nanchang 330006, China

## Abstract

**Background:**

Diabetic cardiomyopathy (DCM) is a disorder of the heart muscle in people with diabetes, which is characterized by both systolic and diastolic dysfunction. The effective treatment strategy for DCM has not been developed.

**Methods:**

Rats were divided into 3 groups with different treatment. The control group was only injected with citrate buffer (n = 8). The diabetes group and diabetes treated group were injected with streptozotocin to induce diabetes. After success of diabetes induction, the rats with diabetes were treated with (diabetes treated group, n = 8) or without (diabetes group, n = 8) recombinant human Neuregulin-1 (rhNRG-1). All studies were carried out 16 weeks after induction of diabetes. Cardiac catheterization was performed to evaluate the cardiac function. Apoptotic cells were determined by TUNEL staining. Left ventricular (LV) sections were stained with Masson to investigate myocardial collagen contents. Related gene expressions were analyzed by quantitative real-time PCR (qRT-PCR).

**Results:**

Diabetes impaired cardiac function manifested by reduced LV systolic pressure (LVSP), maximum rate of LV pressure rise and fall (+dp/dt max and -dp/dt max) and increased LV end-diastolic pressure (LVEDP). The rhNRG-1 treatment could significantly alleviate these symptoms and improve heart function. More TUNEL staining positive cells were observed in the diabetic group than that in the control group, and the rhNRG-1 treatment decreased apoptotic cells number. Furthermore, qRT-PCR assay demonstrated that rhNRG-1 treatment could decrease the expression of bax and caspase-3 and increase that of bcl-2. Collagen volume fraction was higher in the diabetic group than in the control group. Fibrotic and fibrotic related mRNA (type I and type III collagen) levels in the myocardium were significantly reduced by administration of rhNRG-1.

**Conclusion:**

rhNRG-1 could significantly improve the heart function and reverse the cardiac remodeling of DCM rats with chronic heart failure. These results support the clinical possibility of applying rhNRG-1 as an optional therapeutic strategy for DCM treatment in the future.

## Background

Patients with diabetes often develop atherosclerosis and hypertension, both of which are major risk factors to the development of heart disease. However, cardiomyopathy can also be developed in the absence of these established risk factors [1,2]. In the past decades, many studies provided evidences for a specific cardiomyopathy in diabetes (diabetic cardiomyopathy, DCM), which may contribute to myocardial dysfunction in the absence of coronary artery atheroma [3]. DCM is characterized by both systolic and diastolic dysfunction because of reduced contractility, prolonged relaxation, and decreased compliance of the myocardium [4,5]. Pathological mechanism of DCM may be due to myocardial apoptosis and necrosis, reactive hypertrophy, and intermediary fibrosis, structural and functional changes of the small coronary vessels, disturbance of the management of the metabolic cardiovascular load, and cardiac autonomic neuropathy [6]. As so far, there is still no effective treatment strategy for DCM.

Neuregulin-1 (NRG-1) is a widely expressed signaling molecule that is involved in cell differentiation, proliferation, growth, survival, and apoptosis. It is encoded by a large gene (1400 Kb) located in chromosome 8p12, with several promoters and alternative splicing isoforms [7,8]. In adult heart, at least three different NRG-1α isoforms and eight NRG-1β isoforms are expressed. In particular, the β isoform of NRG-1 is highly expressed in the heart and 10 to 100 times more bioactive. NRG-1 expression seems to be restricted to the endothelial cells near cardiomyocytes (in the endocardium and in the myocardial microvasculature), because it is absent in larger coronary arteries, veins and aorta [9]. Hedhli et al. demonstrated that endothelial-derived NRG plays an important role in cardiac myocyte protection against ischemic injury in the heart [10]. Recently, studies using recombinant human neuregulin-1 (rhNRG-1) containing the epidermal growth factor (EGF)-like domain (necessary for ErbB2/ErbB4 activation) demonstrated that NRG-1 plays an important role in heart performance [11,12]. We hypothesized that NRG-1 could improve cardiac function of diabetic rats, probably by regulating cardiac apoptosis and fibrosis, Streptozotocin (STZ)-induced diabetes (Type I) model is well established for investigating DCM in small animals. Therefore, we decided to explore hemodynamic and physiopathological responses to recombinant human NRG-1 (beta isoform, rhNRG-1) in rat DCM model induced by STZ.

## Materials and methods

### Animals and procedures

Experiments were performed in compliance with the ARRIVE guidelines on animal research[13]. Sprague-Dawley (SD) rats at postnatal age of 6 weeks (body weight 200-220 g, Animal center of Nanchang University, Nanchang, China) were assigned to control group (n = 8) and diabetic group (n = 20). Diabetes was induced by intraperitoneal injection of streptozotocin (50 mg/kg; Sigma Chemical, France) [14,15]. Tail vein blood glucose was measured every 3 days in the first week and those with plasma glucose levels ≥16.7 mmol/L were considered to be diabetic. Control rats were injected i.p. with 1 ml/kg body weight of 20 mmol/L citrate buffer (pH 4.5) vehicle at the same time and continued to be raised on standard food and water for the whole experiment period. Twelve weeks after induction of diabetes, 16 diabetic rats remained in the experiment (the rest 4 rats died or ruled out for unsuccessful diabetic induction), which were randomly assigned to the following 2 groups: diabetic rats (citrate buffer injected by tail vein every 2 days during the next 2 weeks, n = 8), and diabetic rats treated with rhNRG-1 (10 μg/kg injected by tail vein every 2 day during the next 2 weeks, n = 8; Novartis Pharmaceutical, Switzerland). The rats were kept on standard food and water for another 2 weeks. All studies were carried out 16 weeks after induction of diabetes.

### Analysis of myocardial function

To evaluate the cardiac function, cardiac catheterization was performed as previously described [16]. Briefly, after the induction of light general anesthesia (anesthetized with 4% chloral hydrate solution (1 ml/100 g) by intraperitoneal injection), one catheter was inserted into the right carotid artery and advanced into the left ventricle (LV). Ventricular pressure signals were measured with a transducer and conditioner (AD instruments MLT0830, Australia) and digitally recorded with a data acquisition system (Power lab, AD instruments, Australia). The following indices were obtained: heart rate (HR), LV systolic pressure (LVSP), LV end-diastolic pressure (LVEDP), and maximum rate of LV pressure rise and fall (+dp/dt max and -dp/dt max). During the process, animals were placed on controlled heating pads. Core temperature was measured via a rectal probe and was maintained at 37°C [17]. Rats were terminated after analysis of myocardial function. The hearts were harvested for the next studies.

### Histopathological process and detection of apoptotic cells by TUNEL staining

Samples were fixed in 10% formalin and paraffin embedded in the surgical pathology facility of Nanchang University. Terminal deoxynucleotidyl transferase-mediated dUTP nick end labeling (TUNEL) analysis was performed with a commercially available kit according to the manufacturer's instructions (Promega, America). Slides were counter stained with hematoxylin (blue). Three midventricular sections (from the apex to the base) of each heart were analyzed. Cardiomyocyte nuclei were quantified by counting randomly 10 fields per section. The apoptotic index (percentage of apoptotic nuclei) was calculated as apoptotic nuclei/total nuclei counted ×100%.

### Analysis of myocardial collagen content

LV sections were stained with Masson to measure interstitial fibrosis. Interstitial collagen was quantified at a final magnification of 200× with a microscope (Olympus BX51, America) connected to a video camera (Nikon DS-Fi1, Japan). Photographs obtained under microscopy were used to calculate the collagen volume fraction of the myocardial interstitium by computer imaging analysis system (MPIAS 500, Japan). The content of interstitial collagen (expressed as the fractional area of the entire cross-section) was averaged on nine fields selected across the wall thickness in the septum and free wall.

### Gene expression analysis by real-time quantitative RT-PCR

Tissue samples obtained from the left ventricle free wall were minced. RNA was extracted as reported before [18]. Total RNA was extracted with Trizol reagent according to the guideline of manufacture (Invitrogen, America) from the samples. For RT-PCR, cDNA was synthesized in a 20 μl reaction volume containing 4 μg of total RNA and Superscript II RT (Fermentas, Canada), according to the instructions of the manufacturer. Real time PCR was carried out using 7500 Real Time PCR System (Applied Biosystems, America), using SYBR Green I (Applied Biosystems, America) as fluorescence dye according to the manufacturer's instructions. Relative quantitation of mRNA expression in the gene of interest was calculated using the comparative threshold cycle number for each sample. To control the variation in the amount of DNA, gene expression of the target sequence was normalized in relation to the expression of an internal control, β-actin. Primers for collagen type, collagen type III, bcl-2, bax, caspase-3 and β-actin were listed as follows:

collagen type I F: 5'- GTTCGTGGTTCTCAGGGTAG -3';

collagen type I R: 5'- TTGTCGTAGCAGGGTTCTTT -3';

collagen type III F: 5'- TGCCCACAGCCTTCTACACCCT -3';

collagen type III R: 5'- CAGCCATTCCTCCCACTCCAG -3';

bcl-2 F: 5'- CGGGAGATCGTGATGAAGT -3';

bcl-2 R: 5'- CCACCGAACTCAAAGAAGG -3';

bax F: 5'- GCAGGGAGGATGGCTGGGGAGA -3';

bax R: 5'- TCCAGACAAGCAGCCGCTCACG -3';

caspase-3 F: 5'-AGTCTGACTGGAAAGCCGAA -3';

caspase-3 R: 5'- CGGGATCTGTTTCTTTGCAT -3';

β-actin F: 5'- TGTGCTATGTTGCCCTAGACTTC -3';

β-actin R: 5'-CGGACTCATCGTACTCCTGCT -3'.

### Statistical analysis

All data were presented as mean ± SEM. Comparisons between groups were made by one-way analysis of variance (ANOVA) with Fisher protected least significant differences post hoc comparison. Results were considered statistically significant if p < 0.05.

## Results

### NRG-1 improved myocardial function

Sixteen weeks after induction of diabetes, rat cardiac function was evaluated by invasive hemodynamic measurements. There was no significant difference in heart rate among the three groups. The lower LVSP and higher LVEDP were observed in diabetic group than that in control group. Resting maximum rates of rise (+dp/dt max) and fall (-dp/dt max) in LV pressure were also impaired after induction of diabetes, indicating that the diabetic rat systolic and diastolic functions were significantly impaired. After the administration of rhNRG-1, the hemodynamic abnormalities mentioned above were remarkably attenuated (Table 1).

**Table 1:** Hemodynamic parameters evaluated by invasive measurements.

Group	HR (beats/min)	LVSP (mm Hg)	LVEDP (mm Hg)	dp/dt (mm Hg/s)	-dp/dt (mm Hg/s)
Control (n = 8)	347 ± 38	131 ± 18	2.1 ± 0.8	6423 ± 417	5723 ± 331
Diabetes(n = 8)	312 ± 29	101 ± 9*	11.4 ± 3.3*	5396 ± 325*	4321 ± 289*
NRG-1(n = 8)	323 ± 32	118 ± 13^#^	6.9 ± 2.4*^#^	5850 ± 387*^#^	4728 ± 302*^#^

Values are presented as mean ± SEM; HR: heart rate; LVSP: left ventricle systolic pressure; LVEDP: left ventricle end diastolic pressure; +dp/dt: maximum rate of left ventricle pressure rise; -dp/dt: maximum rate of left ventricle pressure fall; * P ≤ 0.05 vs. control group; ^# ^P < 0.05 vs. Diabetic group.

### NRG-1 protected cardiomyocytes against apoptosis

**Figure 1:**
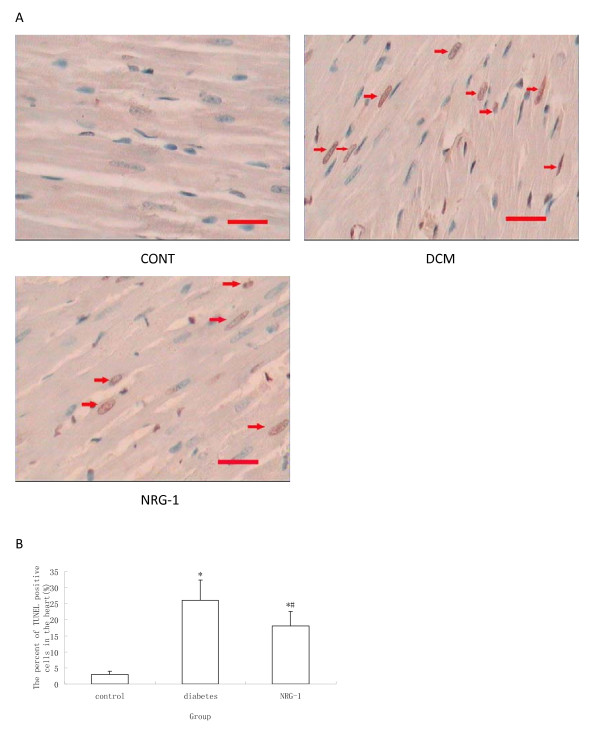
**Analysis of cardiomyocytes apoptosis**. Cardiomyocytes apoptosis detected by TUNEL method. (A) TUNEL staining pictures, in which brown stained cells were TUNEL positive cells, which were defined as "apoptotic". Magnification at 400×, scale bar is 100 μm. (B) Statistical analysis of Cardiomyocytes apoptosis. TUNEL positive cells were increased in Diabetes, and decreased by NRG-1 (n = 8, * P < 0.05 vs. control group; # P < 0.05 vs. Diabetic group.)

**Figure 2:**
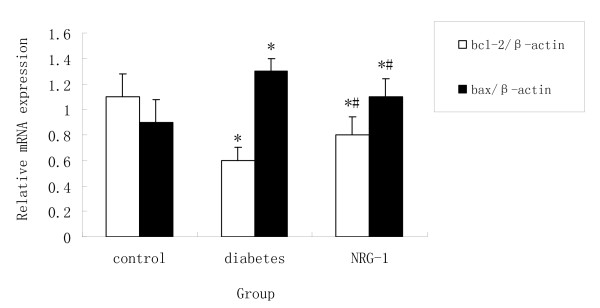
**Statistical analysis of the expression of apoptosis related mRNA detected by PCR**. Compared with Control group, the mRNA expression of Bax was increased and Bcl-2 was decreased in diabetic group, while NRG-1 increased Bcl-2 mRNA expression and decreased Bax mRNA expression when compared with un-treated (Diabetic) group. (n = 8, * P < 0.05 vs. control group; # P < 0.05 vs. Diabetic group.)

**Figure 3:**
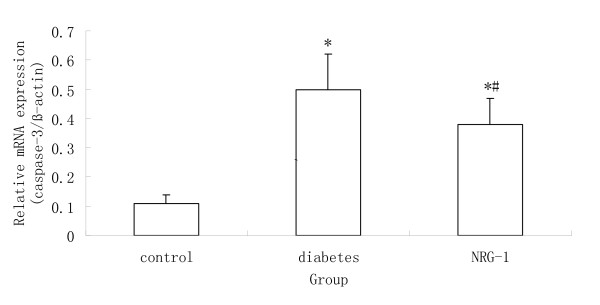
**Statistical analysis of the expression of caspase-3 mRNA detected by PCR**. Compared with Control group, the mRNA expression of caspase-3 was increased in diabetic group, while NRG-1 decreased caspase-3 mRNA expression when compared with un-treated (Diabetic) group. (n = 8, * P < 0.05 vs. control group; # P < 0.05 vs. Diabetic group.)

TUNEL assay was performed to examine the apoptosis *in vivo*. The numbers of TUNEL staining positive cells in the diabetic group were higher than that in the control groups (Figure 1). The rhNRG-1 treatment resulted in significantly less apoptotic cells than untreated group. Quantitative real-time PCR was used to determine the mRNA expression of bcl-2, bax, and capase-3, which are regarded as the markers of apoptosis. It was showed that bcl-2 was down regulated but bax and capase-3 were up regulated in the diabetic group, whereas, rhNRG-1 alleviated the changes (Figure 2, 3). Since bcl-2 is known for anti-apoptosis, and bax and caspase-3 pro-apoptosis, these results indicate that rhNRG-1 treatment could protect cardiomyocytes against apoptosis.

### NRG-1 attenuated myocardial interstitial fibrosis

**Figure 4:**
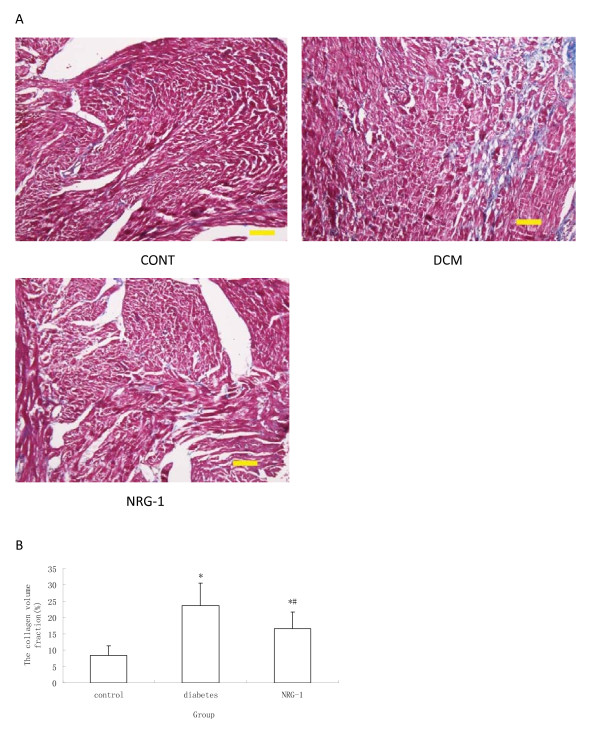
**Analysis of interstitial fibrosis**. (A) Fibrotic infiltration in the myocardium with Masson's trichrome staining. Area stained blue represent fibrotic infiltration. Magnification at 200×, scale bar is 100 μm. (B) Quantitative analysis of fibrosis. The collagen volume fraction was higher in the diabetic group than in the control group, while NRG-1 attenuated such increase. (n = 8, * P < 0.05 vs. control group; # P < 0.05 vs. Diabetic group.)

**Figure 5:**
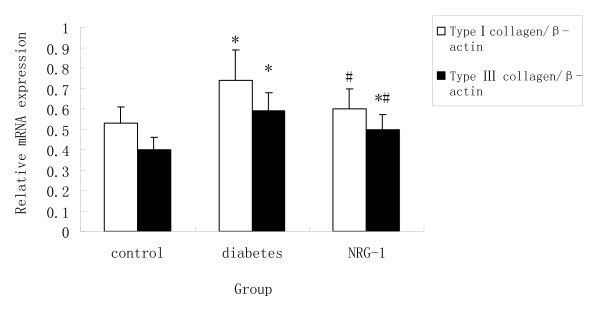
**Statistical analysis of the expression of collagen type I (A) and type III (B) mRNA by PCR**. Compared with Control group, the mRNA expression of collagen type I and type III was increased in diabetic group, while NRG-1 suppressed such induction of collagen type I and type III mRNA. (n = 8, * P < 0.05 vs. control group; # P < 0.05 vs. Diabetic group.)

The collagen volume fraction, which is an indicator of interstitial fibrosis, was higher in the diabetic group than in the control group (Figure 4). Similarly, the type I and type III collagen mRNA expression levels were also significantly up regulated in the diabetic group (Figure 5). These fibrotic and fibrotic related genes over-expression in the myocardium was remarkably inhibited by the administration of rhNRG-1, suggesting that rhNRG-1 treatment could attenuate myocardial interstitial fibrosis caused by diabetes.

## Discussion

Our data suggests a fresh optional strategy for DCM treatment. Through our studies with rhNRG-1 in DCM rats, we found that the administration of NRG-1 could improve cardiac function and reversed remodeling of the heart of DCM rats by regulating cardiomyocytes apoptosis and cardiac fibrosis. The data also further supports the hypothesis that NRG-1 plays an important role in regulating heart function.

NRG-1 acts as a paracrine factor via the ErbB family of tyrosine kinase receptors expressed in cardiomyocytes. The ErbB-receptors are a family of four transmembrane receptors that bind multiple growth factors, including epidermal growth factor (EGF), transforming growth factor-α (TGF-α), and NRG1-4. ErbB3 is expressed in prenatal myocytes, whereas, adult ventricular myocytes express only ErbB1, ErbB2, and ErbB4. It is known that ErbB1, also known as the EGF-receptor, does not bind NRG-1, so only ErbB2 and -4 do serve as NRG-1's receptors in adult cardiomyocytes [19,20]. NRG-1 acts through ErbB2 and ErbB4 in a paracrine fashion to stimulate MEK/ERK, Akt/PI3-kinase, Src/FAK, and NO synthase, which together promote myocyte function and survival in the setting of cardiac stress [21-24]. Recently, hNRG-1 was successfully used to improve the preservation of rodent heart. The functional improvement was accompanied by increase phosphorylation of Akt, ERK, STAT3 and GSK- 3β[25]. NRG-1 stimulates activation of MEK/ERK with subsequent induction of protein synthesis and hypertrophic gene expression. Another downstream signaling pathway is the Akt/PI3-kinase pathway which appears to be involved in the protection of cardiac myocytes against cell death, as well as regulation of metabolism and growth. NRG-1 induces PI3-kinase dependent activation of Akt in cardiac myocytes, a pathway that has been heavily implicated in cell survival [26,27]. Interestingly, Akt-GSK-3β pathway has been shown to be important in mediating the cardioprotective effects of tanshione IIA on STZ induced diabetic cadiomyopathy in rats [6]. Increased cardiomyocyte apoptosis has been described in hearts of diabetic mice and patients, and hypothesized as an important mechanism of DCM [28]. Our results suggest that anti-apoptosis effect may contribute to myocardial protection of NRG-1[6].

DCM is considered as interstitial and perivascular fibrosis. It was known that myocardial fibrosis could cause myocardial dysfunction in diabetes. Our findings also showed that NGR-1 attenuated heart fibrosis. The precise mechanism was unclear. Recent study showed that TGF-β could contribute fibrosis by binding to ErbB-receptors [29]. Enhancements of NRG-1 may compete with TGF-β by binding to ErbB-receptors to lessen the fibrosis triggered through the TGF-ErbB signal pathway. NO synthase, which can be stimulated by NRG-1/ErbB signal pathway, may also be involved in myocardial fibrosis. It was reported that NO synthase could attenuate myocardial fibrosis by regulating renin release [30]. Up-regulation of the renin-angiotensin system (RAS) has been described in diabetes and is associated with development of cardiac hypertrophy and fibrosis. In addition, cardiomyocytes and endothelial cells in the hearts of individuals with diabetes and end-stage heart failure manifest evidence of oxidative stress, apoptosis, and necrosis that correlate with RAS activation [2]. In the present study, it is highly possible that some part of the actions NRG-1 inhibiting cardiac remodeling is through the regulation of rein release through NO synthase.

It was reported that NRG-1/ErbB expression declined in the later stages when pump failure occurred [31], The decline in NRG-1 expression coincided with the development of eccentric ventricular hypertrophy and pump failure and was accompanied by a down regulation in the mRNA levels of ErbB2 and ErbB4. Presumably, it is due to the increased levels of angiotensin II and epinephrine, both of which reduce NRG-1 mRNA synthesis in cardiac endothelium [32]. The replenishment of NRG-1 may inhibit the physiopathological aggravation of DCM.

## Conclusion

The heart function of DCM rats with chronic heart failure was significantly improved by NRG-1, supporting the rationale of replenishing NRG-1 as an optional selection for DCM therapy, although further clinical studies are required. rhNRG-1 seems to be able to protect cardiomyocytes against apoptosis and reduce the extent of myocardial interstitial fibrosis. The exact mechanism will be pursued in the future studies.

## List of abbreviations

DCM: diabetic cardiomyopathy; rhNRG-1: recombinant human neuregulin-1; EGF: epidermal growthfactor; STZ: streptozotocin; SD: Sprague-Dawley; LV: left ventricle; HR: heart rate; LVSP: LV systolicpressure; LVEDP: LV end-diastolic pressure; +dp/dt: maximum rate of left ventricle pressure rise; -dp/dt: maximum rate of left ventricle pressure fall; SEM: standard error of mean.

## Competing interests

The authors declare that they have no competing interests.

## Authors' contributions

All authors fulfill the criteria for authorship. BL, ZZ and YW designed and drafted the protocol, MWand JP contributed to the design and coordination of the study, XH and JX contributed to the statistical analysis and coordination of the study, TK, YL and ZL interpreted the findings and drafted the manuscript. All authors read and approved the final manuscript.
